# Insight into shark magnetic field perception from empirical observations

**DOI:** 10.1038/s41598-017-11459-8

**Published:** 2017-09-08

**Authors:** James M. Anderson, Tamrynn M. Clegg, Luisa V. M. V. Q. Véras, Kim N. Holland

**Affiliations:** 1 Hawai’i Institute of Marine Biology, University of Hawai’i at Mānoa, Kāne’ohe, HI 96744 USA; 2 Department of Biology, University of Hawai’i at Mānoa, Honolulu, HI 96822 USA; 3 Laboratório de Oceanografia Pesqueira, Universidade Federal Rural de Pernambuco, Recife, 52171-900 Brazil; 4 School of Ocean and Earth Science and Technology, University of Hawai’i at Mānoa, Honolulu, HI 96822 USA

## Abstract

Elasmobranch fishes are among a broad range of taxa believed to gain positional information and navigate using the earth’s magnetic field, yet in sharks, much remains uncertain regarding the sensory receptors and pathways involved, or the exact nature of perceived stimuli. Captive sandbar sharks, *Carcharhinus plumbeus* were conditioned to respond to presentation of a magnetic stimulus by seeking out a target in anticipation of reward (food). Sharks in the study demonstrated strong responses to magnetic stimuli, making significantly more approaches to the target (*p* = < 0.01) during stimulus activation (S+) than before or after activation (S−). Sharks exposed to reversible magnetosensory impairment were less capable of discriminating changes to the local magnetic field, with no difference seen in approaches to the target under the S+ and S− conditions (*p* = 0.375). We provide quantified detection and discrimination thresholds of magnetic stimuli presented, and quantify associated transient electrical artefacts. We show that the likelihood of such artefacts serving as the stimulus for observed behavioural responses was low. These impairment experiments support hypotheses that magnetic field perception in sharks is not solely performed via the electrosensory system, and that putative magnetoreceptor structures may be located in the naso-olfactory capsules of sharks.

## Introduction

Many animals from several taxa are able to perceive the earth’s magnetic field and discriminate changes in that field^1^. The ability to detect and orient to magnetic fields has been observed in bacteria^2, 3^, algae^4^, invertebrates^5–7^ and vertebrates, including birds^8–11^, rodents^10, 12^, amphibians^13–15^, quelonians^16, 17^, cetaceans^18^ and teleost fish^19–27^. Geo-magnetic field parameters such as field direction, field vector (horizontal & vertical components), inclination, declination & magnitude/intensity could all provide positional information. The characteristics/parameters of the magnetic field that are perceived by organisms have been found to vary across taxa (Table 1). Elasmobranch fishes have been both hypothesised^28–30^ and empirically shown to respond to changes in magnetic fields^31–34^. Certainly, several species make large scale movements that could be assisted by even coarse-scale perception of magnetic fields^35–38^. However, the sensory pathways responsible for mediation of elasmobranch magnetic field perception have yet to be definitively identified

**Table 1:** Taxa demonstrated to perceive or respond to magnetic field changes.

Systematic Group	Perceived Magnetic Parameter	Reference
**Proteobacteria**		
*Magnetotactic bacteria*	Polarity (compass)	3, 76
**Euglenophyta**		
*Magnetotactic algae*	Polarity (compass)	4
**Mollusca**		
*Snail*	Polarity (compass)	77
**Arthropoda**		
*Spiny Lobster*	Vector (compass)	6
*Honey bee*	Intensity (map)	7
**Chordata (Vertebrata)**		
*Newt*	Vector (compass)	14
*Salamander*	Inclination (compass)	78, 79
*European Robin*	Inclination (map)	9
*Homing pigeon*	Polarity (compass)	60, 62, 65, 66
*Mole Rat*	Polarity (compass)	12
*Eel*	Polarity (compass)	19
*Turtle*	Inclination (map)	16, 17
*Cetacean*	Intensity (map)	18
*Salmon*	Inclination (map)	26, 27
*Trout*	Intensity (map)	23, 24
*Tuna*	Intensity (map)	22
*Elasmobranch*	Polarity (compass)	33

Table provides examples of taxa across different phyla, the magnetic field parameter perceived, as well as the orientational or navigational step associated with the magnetic field parameter perceived.

The mechanisms by which sharks may perceive and use magnetic information have been the subject of continuing debate. Kalmijn^31, 33, 39^ proposed elasmobranch magnetoreception may be facilitated through the electrosensory system, which transduces magnetic field components into electrosensory stimuli. Later refinement of the electromagnetic induction theory by Paulin^28^ set out a feasible mechanism by which elasmobranchs could perceive the geomagnetic field. Paulin argued that based on the work of Montgomery^40^, electroreceptors do not have the characteristics necessary to make DC measurements, because the rapid desensitization of electroreceptors to a DC field would preclude detection of electromotive fields in the manner suggested by Kalmijn. Alternatively, a directional compass sense may be facilitated by a comparison of inputs from the vestibular and electrosensory systems, making use of directional asymmetry in the voltage drop across electroreceptors during movement of the head^28^. That is, as the animal swims, the head moves side to side in a sinusoidal manner, producing phasic/cyclical stimuli to sensory maculae within the vestibular system, as well as a voltage drop across the ampullae of Lorenzini corresponding to the change in head position relative to the external electrical field as the animal moves. These common-mode electrosensory stimuli (i.e. they occur across all electrosensory afferents) are suppressed or eliminated by an adaptive filter or common mode suppression mechanism in the dorsal octavolateral nucleus of the shark brain^41, 42^, allowing a high signal to noise ratio. In-fact, it has been demonstrated that even artificial common-mode stimuli are cancelled by the adaptive filter mechanism^42^. Sharks could maintain a constant swimming direction relative to the geomagnetic field by maintaining a constant electrosensory “chord”, comprised of different amplitudes at the harmonics of the vestibular frequency^43^. Via this electrosensory-vestibular hypothesis, receptor field projection could facilitate discrimination of changes to the polarity (north-south directionality) of geomagnetic fields (relative to the shark) as the animal moves either across or along geomagnetic field lines (assuming movement in a relatively constant horizontal plain). This has yet to be empirically shown.

Behavioural studies in elasmobranchs that have provided empirical evidence for magnetic field perception have been criticised^44, 45^ for failing to sufficiently control for the possibility that sharks were responding to transient electrical artefacts caused by activation of experimental magnetic stimuli rather than changes in the ambient magnetic field *per se* ^32^, or that magnetosensory impairment methods used^46^ may have similarly resulted in induced electrical field stimuli that could possibly impact an induction-based magnetosensory system. The bulk of subsequent studies into the ability of elasmobranch’s to perceive magnetic fields have used lanthanide metals (‘rare earth’ metals with strong magnetic properties) as potential bycatch deterrents in fisheries^47–52^. These studies have shown that different metals within the lanthanide series have differing and species–specific efficacies as a repellent, and these impacts are generally minimal, transitory and context-specific. This latter point refers to the fact that behavioural responses are impacted by hunger motivation and the number of animals involved in the test. Most recently, Newton and Kajiura^53^ reported that yellow stingrays (*Urobatis jamaicensis*) could be trained to locate and feed over specific neodymium magnets in behavioural choice test experiments, which demonstrates that these metals have no intrinsic repellent properties. An exception to this came from a study using adult sandbar sharks (n = 3) in individual feeding trials that determined neodymium magnets could be effective as a repellent^54^. Animals in the study were not fasted prior to testing.

Here we present the results of conditioned behaviour experiments conducted with juvenile sandbar sharks *Carcharhinus plumbeus* (Nardo, 1827), designed to provide direct empirical evidence of magnetic field perception by introducing localised interference in ambient magnetic fields. Reversible sensory impairment methods were used to test the hypothesis that receptors for the perception of magnetic stimuli may be located in the naso-olfactory capsules of sharks. This hypothesis invokes the existence of magnetosensory structures within the olfactory organs, perhaps homologous to systems described in some teleost and avian species^23, 46, 55^. In the experiments reported here, the nature of the magnetic and electrical fields in the test arena were known. Given these experimental conditions, we attempt to narrow the possibilities regarding which sensory pathways are involved in magnetic field perception, and whether magnetic fields *per se* (rather than an electrical derivative) are detectable by sharks.

## Results

### Responses to magnetic stimuli

Sharks were successfully conditioned to respond to the presentation of a magnetic stimulus (S+). Unimpaired, conditioned sharks produced a 100% response (all sharks demonstrating a conditioned response) to all applied magnetic field intensities used in training and conditioning, which ranged from 0.03 to 2.89 micro-Tesla (μT). Conditioned sharks reacted by increasing tail beat frequency and swim speed, then converging on the target in anticipation of receiving a food reward.

**Figure 1:**
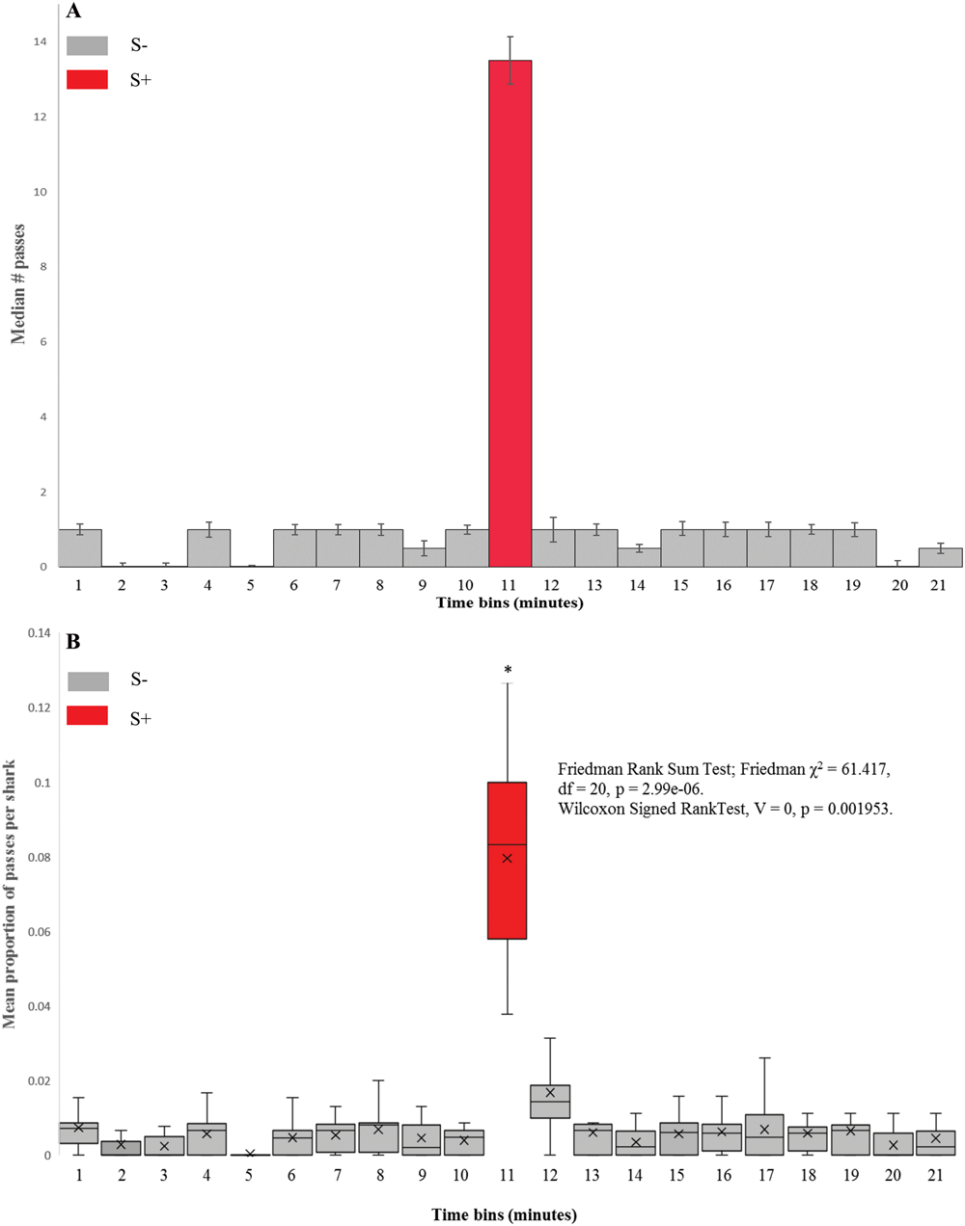
Behavioural responses of unimpaired sharks to presented magnetic stimulus. (**A**) Histogram showing median number of passes over the target for all sharks, in each 1 minute time bin. S+ minute is shaded red, all other 1 minute time bins (S−) are shaded grey. Error bars show standard error. (**B**) Box & Whisker plot showing mean proportion of passes over the target, per shark, across the series of ten unimpaired trials, mean value is denoted by x. S+ minute is shaded red, all other 1 minute time bins (S−) are shaded grey. Friedman Rank Sum tests were used to determine any differences in time bins. Wilcoxon Signed Rank Tests were used to compare mean proportion of passes over the target, averaged per shark in the eleventh minute time bin with each other one minute time bin. Averaged per shark, a significantly higher proportion of passes over the target was seen in the S+ (11th) minute. *p < 0.01.

In test trials (food reward not given), the number of passes over the target by all animals combined during the S+ minute ranged between 6 and 19, with a median pass rate of 13.5 ± 3.8 (Fig. 1A). By comparison, median number of passes for all S− one minute bins across all 10 trials ranged from 0–2. The median number of passes for all other one minute time bins combined under ‘control’ (non-stimulus) conditions was 1 ± 0.89. The number of sharks used across the series of 10 unimpaired trials did not remain constant. 5 animals were used for the first eight trials, 7 animals were used in the final two trials. The number of passes across the target in the S+ minute during the final two trials in this series did not increase as a result of more sharks being present. The 95% confidence intervals for the control group were 0.0589 and 0.100. One data point fell above this interval, from a trial with five sharks. Three data points fell below this range (i.e. proportion of responses, averaged per shark was low) (Table 2). One of these data points came from a trial using five sharks, the other two came from the two trials using seven sharks (Table 2). Proportion of passes over the target, averaged per shark, during the S+ minute were significantly greater than in any other one minute time bin (Fig. 1B, Friedman Rank Sum Test; Friedman χ^2^ = 61.417, df = 20, p = 2.99e^−06^, Wilcoxon Signed Rank Test, Test, V = 0, p = 0.001953.

**Table 2:** Proportion of passes over the target, per shark, across the series of ten unimpaired trials.

Trial #	**1**	**2**	**3**	**4**	**5**	**6**	**7**	**8**	**9**	**10**
# Sharks	5	5	5	5	5	5	5	5	7	7
**Minute**										
1	0	0.009	0.008	0.006	0	0.015	0.02	0.008	0.004	0.003
2	0	0	0.004	0	0	0.015	0.007	0	0	0.003
3	0	0	0.004	0	0	0.008	0.007	0	0	0.005
4	0.007	0.009	0	0	0	0.008	0.007	0.017	0	0.011
5	0	0	0	0	0	0	0	0	0	0.003
6	0.007	0	0.004	0	0	0.015	0.007	0	0.009	0.005
7	0.007	0.009	0	0.006	0	0.008	0	0.008	0.013	0.003
8	0.02	0.009	0	0.013	0	0.008	0	0.008	0.009	0.003
9	0	0	0.004	0.013	0	0.008	0	0	0.013	0.008
10	0.007	0.009	0.008	0.006	0	0	0	0	0.004	0.005
**11**	**0.127**	**0.07**	**0.063**	**0.097**	**0.1**	**0.046**	**0.1**	**0.1**	**0.056**	**0.038**
12	0.013	0	0.031	0.019	0.044	0.015	0.013	0.017	0.009	0.005
13	0.007	0.009	0.008	0.006	0	0.023	0	0.008	0	0
14	0	0	0	0	0.011	0.008	0.007	0	0.004	0.005
15	0	0.009	0.016	0.013	0	0.008	0	0	0.004	0.008
16	0.007	0.009	0.016	0.006	0	0.015	0	0	0.004	0.005
17	0	0.026	0.012	0	0	0	0.013	0.008	0.004	0.005
18	0	0.017	0.008	0.006	0.011	0	0.007	0	0.004	0.005
19	0	0	0.008	0.006	0.011	0	0.007	0.025	0	0.008
20	0	0	0.008	0	0.011	0	0	0	0	0.008
21	0	0.017	0	0	0.011	0	0.007	0	0.004	0.005

The number of sharks tested in each trial is shown. 95% confidence intervals were 0.0589 and 0.100. S+ (11^th^) minute is highlighted in **bold**.

### Responses to magnetic stimuli following sensory impairment

**Figure 2:**
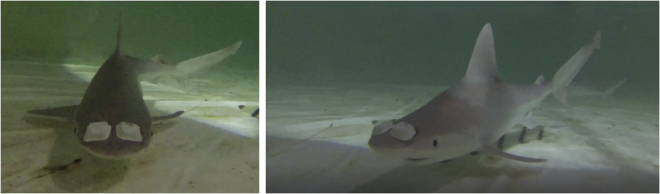
Placement of magnets designed to impair magnetic stimulus perception. Neodymium magnets were embedded horizontally in gelatine within a sealed container and aligned with the longitudinal axes of the olfactory organs.

We attempted to induce magnetosensory impairment in sharks by reversibly attaching magnets to the dorsal surface of the head (Fig. 2). The test animals showed no adverse responses to the manipulations involved with attachment and removal of the sensory impairment devices. They swam in a normal fashion, and fed readily when presented with the food reward in training/conditioning. Sharks that had undergone impairment treatment successfully produced the CR in as little as thirty minutes following application of the impairment devices. A strong conditioned response continued to be evoked during training and reinforcement across the duration of the impaired trial series. Thus, the physical manipulation of the animals and attachment of the magnets was judged not to impair conditioned feeding responses.

In initial impairment testing, sharks again produced the conditioned response (faster swimming, elevated tailbeat frequency, convergence upon the target) upon presentation of these higher magnetic stimuli. Notably, the magnitude of the conditioned response decreased when the magnetic stimulus presented was reduced (Table 3).

**Table 3:** Conditioned behavioural responses of sensory-impaired sharks to presentation of stronger magnetic stimuli.

Trial no.	1	2	3	4	5
*Β* field (µT)	8	8	5.3	1.2	1.4
No. sharks	5	5	5	5	5
**Minute**	**Passes over target**				
1	1	0	5	1	1
2	3	0	2	1	1
3	0	2	1	2	1
4	0	1	1	1	2
5	1	0	4	0	2
6	0	1	0	1	4
7	0	2	3	2	2
8	1	1	4	2	1
9	1	3	3	2	2
10	2	0	2	3	2
**11**	**9**	**12**	**8**	**5**	**3**
12	4	2	2	0	1
13	2	1	1	3	1
14	2	2	1	1	2
15	0	1	3	1	1
16	0	3	2	1	1
17	1	0	2	0	1
18	1	0	1	3	0
19	1	1	2	0	5
20	2	0	5	2	2
21	0	1	1	2	2

Counts of passes over the target, summed for all sharks (n = 5), for each minute of each trial are shown. S+ (11^th^) minute is highlighted in **bold**.

**Figure 3:**
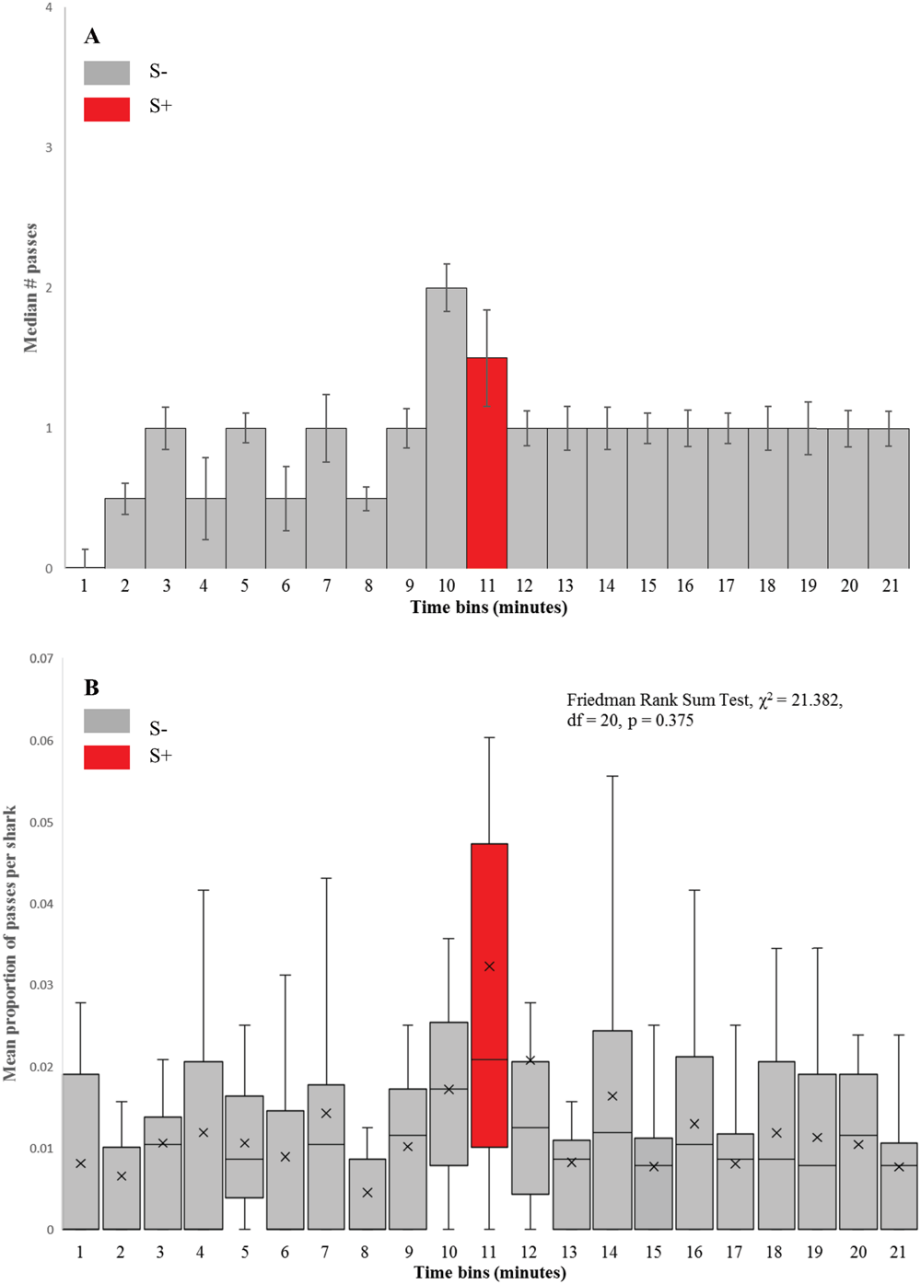
Behavioural responses of sensory impaired sharks to presented magnetic stimulus. (**A**) Histogram showing median number of passes over the target for all sharks, in each 1 minute time bin. S+ minute is shaded red. Error bars show standard error. (**B**) Box & Whisker plot showing mean proportion of passes over the target, averaged per shark, across the series of ten sensory-impaired trials, mean value is denoted by x. S+ minute is shaded red, all other 1 minute time bins (S−) are shaded grey. Friedman Rank Sum tests were used to determine any differences in time bins. No difference was found under S+ or S− conditions when animals had undergone magnetic impairment treatment.

To compare the conditioned responses of control/normal and impaired sharks, magnetic stimuli of ecologically relevant magnitudes (0.03 μT) were applied to the experimental arena. Four sharks were used in seven of the ten trials, three in the remaining three trials. In all cases the CR evoked from impaired animals when presented with the 0.03 µT stimulus was markedly diminished when compared to sharks without sensory impairment. Increases in tailbeat frequency were visibly smaller and a faster return to ‘normal’ background behaviour was also observed in impaired animals. However, the magnitude of the conditioned response during impairment testing was variable (range = 0–7 passes per trial). In these tests, both the highest and lowest number of passes across the target occurred in trials with four animals. No significant difference was seen in the overall response in the S+ minute across all ten trials in this impaired series (Friedman Rank Sum Test, χ^2^ = 22.765, df = 20, p = 0.301). The median pass rate in the S+ minute was 2 ± 2.4, median pass rate for all other time bins was 1 ± 0.98 (Fig. 3A). No difference was found in the proportion of passes, averaged per shark, over the target between the S+ and S− conditions (Fig. 3B, Friedman Rank Sum Test, χ^2^ = 21.382, df = 20, p = 0.375). The 95% confidence intervals were 0.012 and 0.059. Four data points fell below this range, two from trials using three sharks, two from trials using four sharks (Table 4). One data point was above this range (four shark trial). The remaining five trials fell within the confidence interval (Table 4). The proportion of passes over the target, averaged per shark, in the S+ minute of the unimpaired/normal series of trials was found to be significantly greater than in the S+ minute of the sensory-impaired series (Wilcoxon Ranked Sum Test, W = 85, p = 0.009).

**Table 4:** Proportion of passes over the target, per shark, across the series of ten impaired trials.

Trial #	**1**	**2**	**3**	**4**	**5**	**6**	**7**	**8**	**9**	**10**
# Sharks	5	5	5	5	5	5	5	5	7	7
**Minute**										
1	0.000	0.023	0.000	0.000	0.017	0.021	0.000	0.028	0.000	0.000
2	0.000	0.011	0.016	0.009	0.009	0.000	0.000	0.028	0.000	0.000
3	0.012	0.011	0.016	0.009	0.000	0.021	0.010	0.000	0.038	0.000
4	0.024	0.000	0.039	0.009	0.017	0.000	0.042	0.000	0.000	0.000
5	0.012	0.023	0.008	0.009	0.009	0.021	0.010	0.000	0.025	0.000
6	0.012	0.011	0.031	0.026	0.017	0.000	0.000	0.000	0.000	0.000
7	0.012	0.023	0.000	0.043	0.000	0.000	0.010	0.000	0.013	0.056
8	0.012	0.000	0.008	0.009	0.009	0.000	0.000	0.000	0.013	0.000
9	0.012	0.011	0.008	0.017	0.017	0.000	0.021	0.000	0.025	0.000
10	0.036	0.023	0.016	0.017	0.017	0.021	0.031	0.028	0.000	0.000
**11**	**0.036**	**0.011**	**0.039**	**0.009**	**0.060**	**0.021**	**0.000**	**0.056**	**0.013**	**0.111**
12	0.024	0.011	0.016	0.009	0.017	0.000	0.000	0.028	0.013	0.111
13	0.036	0.011	0.016	0.009	0.009	0.000	0.010	0.000	0.000	0.000
14	0.012	0.034	0.000	0.009	0.000	0.021	0.021	0.028	0.000	0.056
15	0.012	0.000	0.008	0.000	0.009	0.021	0.010	0.000	0.025	0.000
16	0.000	0.011	0.000	0.009	0.017	0.042	0.010	0.028	0.025	0.000
17	0.012	0.011	0.000	0.009	0.000	0.021	0.010	0.000	0.025	0.000
18	0.024	0.034	0.008	0.009	0.017	0.000	0.010	0.028	0.000	0.000
19	0.000	0.034	0.008	0.017	0.000	0.021	0.031	0.000	0.013	0.000
20	0.024	0.011	0.008	0.017	0.000	0.021	0.021	0.000	0.013	0.000
21	0.024	0.023	0.008	0.009	0.009	0.000	0.000	0.000	0.013	0.000

The number of sharks tested in each trial is shown. 95% confidence intervals were 0.012 and 0.059. S+ (11^th^) minute is highlighted in **bold**.

### Electrical field measurement and calculation

**Figure 4:**
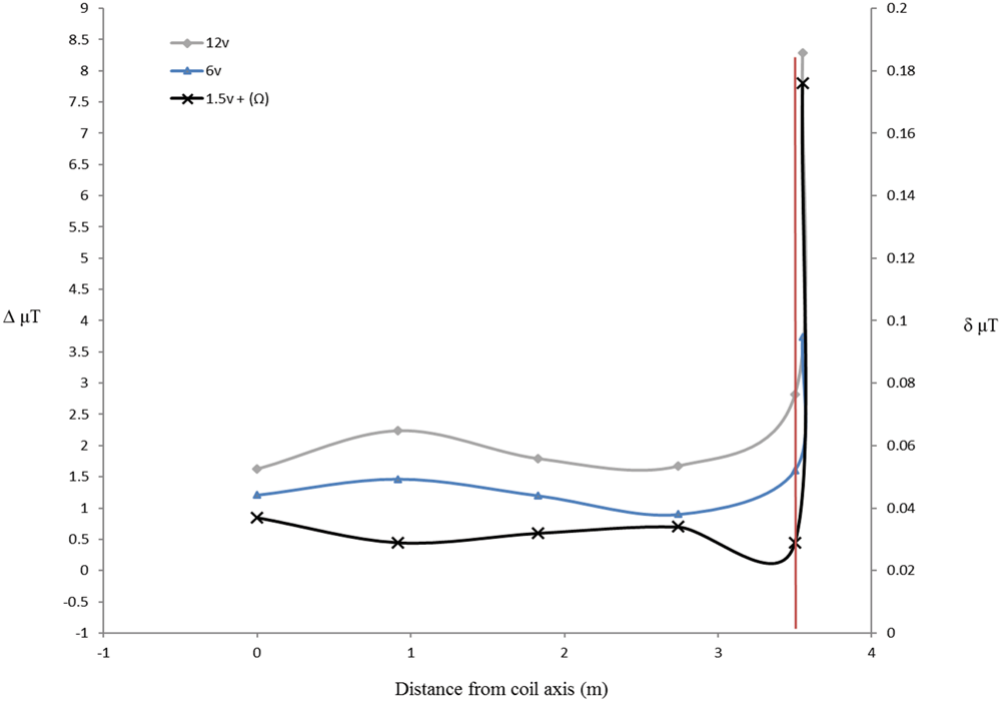
Measured profiles of total magnetic intensity. Changes in magnetic field intensity (μT) associated with magnetic stimulus presentation were measured across the diameter of the tank at increments of 3 ft (0.9144 m), from centre (0 m) to periphery (3.5 m) (coil axis is the centre of the tank). Y axis values correspond to magnetic field changes (Δ µT) associated with use of 12 volt and 6 volt power sources. Z axis values correspond to magnetic field changes (δ µT) associated with use of 1.5 volt power source, with 5.6k Ω of resistance built into the circuit. Vertical red line indicates tank periphery.

**Figure 5:**
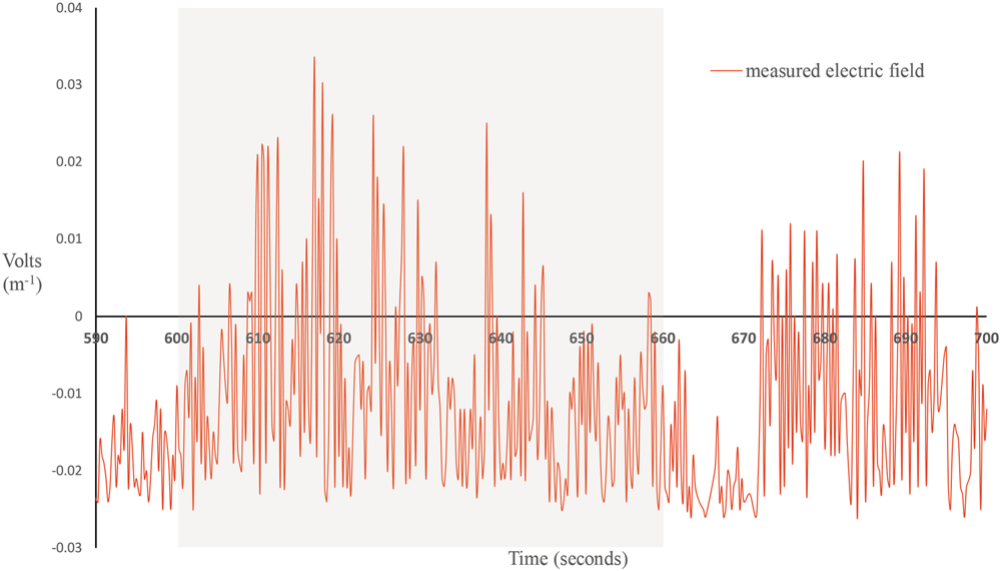
Electrical field measurements associated with background electrical noise. Electrical field artefacts occurring during presentation of the magnetic stimulus were measured using a Trifield® Natural EM Meter (AlphaLab Inc., Salt Lake City, UT, USA), capable of taking measurements every millisecond. Grey shaded region indicates period of magnetic field activation (S+ minute; 600–660 seconds). *N.b*. the constant and random fluctuation of background electrical noise in the environment, both before, during and after magnetic field activation. Maximum range of recorded background electrical field oscillation (noise) during the period of stimulus activation was 51 mV, occurring at rate of 1.02 mV/cm/s^−1^. Peak voltage (maximum “spike” above zero) was 33 mV, or 3.3e^7^ nV which occurred 17 seconds after stimulus activation.

**Figure 6:**
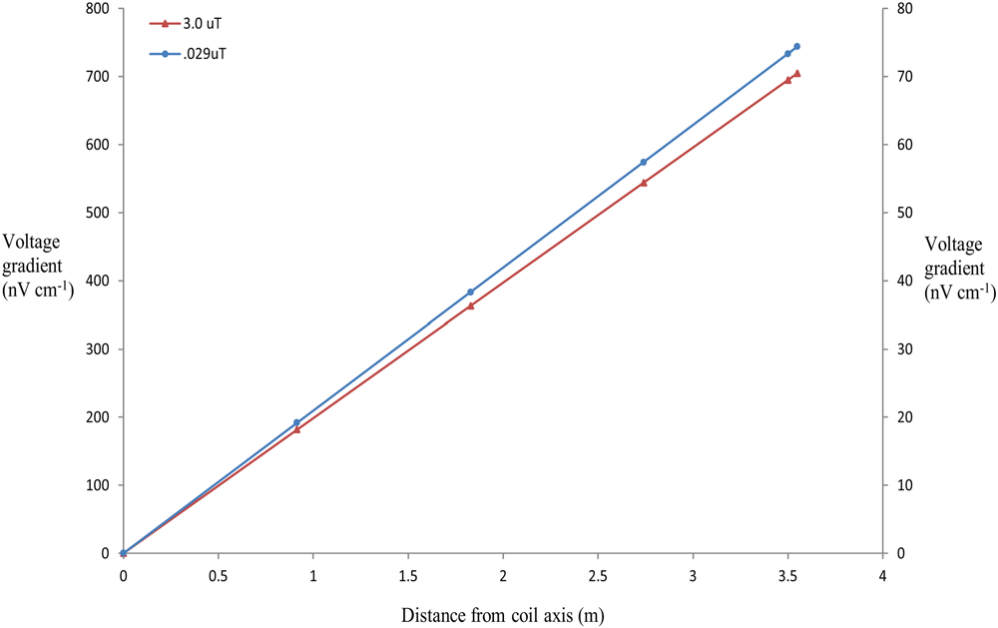
Calculated induced voltage gradient associated with magnetic stimulus. Modelled gradients correspond to changes in total magnetic field intensity of 3 μT (red line) and 0.029 μT (blue line) respectively. Red line corresponds to Y axis, blue line corresponds to Z (secondary) axis. Voltage gradients induced by modification of the local magnetic field within the tank were calculated from centre (coil axis – 0 m) to periphery (3.5 m) at increments of 3 ft (0.9144 m). Induced voltages increased linearly with distance, peaking at 73.3 nV cm^−1^ at the tank periphery when a magnetic field modification of 0.029 µT was applied. Calculated time to reach peak induced voltage gradient following onset of magnetic stimulus was 2.7 milliseconds.

The magnetic field within the tank varied according to the current applied (Fig. 1). Incorporation of a 5.6k ohm (Ω) resistor in the circuit resulted in small and more uniform magnetic field across the diameter of the tank. A 1.5 v applied charge combined with the 5.6k Ω resistor resulted in a magnetic field generation of 0.029 μT above the local field (Fig. 4). Electric field measurements taken with a Trifield^®^ Natural EM Meter before, during and after circuit activation revealed a variable background electrical field oscillation with a range as high as 51 milliVolts per metre (mV/m^−1^) occurring over a period 0.5 seconds (Fig. 5). Thus, background electrical flux was as high as 1 mV/cm/s^−1^. Transient electrical artefacts induced by changing the magnetic field occurred within this range of background noise, and thus were undetectable using the meter. Subsequent efforts to measure transient induced voltage gradients using electrophysiological equipment also proved unsuccessful, due to the complexity of background electrical noise (Fig. 5). Thus, we calculated the magnitude of any transient voltage gradient using a combination of Faraday’s Laws and Maxwell’s equations (see Methods). Calculated transient voltage gradients were highest at the periphery of the tank and weakest (zero) in the centre (Fig. 6). Under application of weak magnetic fields (0.029 μT) used in testing, the maximum transient voltage gradient was calculated to be 74.35 nanoVolts (nV) cm/s^−1^ (Fig. 6) at the periphery of the tank. This induced electric field decays as a function of the inverse cube of the distance from the coil, but our calculations indicate that it remained above the 30 nV cm^−1^ median sensory threshold across more than half of the experimental arena (Fig. 6). The time taken for the current flowing in the circuit to reach a steady state, thus the duration of a transient voltage gradient within the experimental arena, was 2.7 milliseconds. We observed 100% response to presentation of magnetic fields of this magnitude (0.029 μT) in unimpaired sharks.

## Discussion

In their 2005 review, Johnsen and Lohmann^45^ suggested that conditioned-behaviour magnetic field experiments such as those of Meyer *et al*.^32^ could not unequivocally claim that shark responses were to magnetic stimuli *per se* because it was possible that the activation of a Helmholtz coil (or similar magnetic field generation method) would produce a transient electrical field that could be detected by the electrosensory receptors of the test animals. They similarly argued that experiments reported by Walker^46^ may also have been influenced by induced electrical effects that were not accounted for. In the current experiment, we have quantified the background ambient electric field environment and calculated transient voltage gradients induced through activation of an altered magnetic environment. All sharks used in the study (normal and impaired conditions) were fasted for 24 hours before testing, and were not fed during test trials. Thus, motivation to feed would not be expected to be a factor that might alter the response of the animals under presentation of different strengths of magnetic stimuli used in training and in testing.

In the experiments reported here, we used a single coil with 100 loops to introduce an altered magnetic field within the tank. Electrical field tests using both a field probe and an electrophysiological recording set-up, conducted within the tank from the periphery to centre at intervals of three feet (91 cm), were unable to register a transient voltage gradient during activation of the stimulus coil. This was largely due to complex and fluctuating background electrical noise (Fig. 5). In the test arena used in the study, electrical noise fluctuated unpredictably, and fell within a range of 0 to 51 mV (Fig. 5). Empirical investigation has found the minimum voltage required to elicit any behavioural response in C. *plumbeus* was found to be 0.5 nV cm^−1^, while the median behavioural response threshold for C. *plumbeus* was found to be 30 nV cm^−1^ ^56^. The median sensitivity to electrical stimuli reported across elasmobranch species is 35 nV cm^−1^ ^57^.

We calculated the magnitude of possible transient electrical field artefacts at different points in the tank using the different magnetic field strengths applied in both training and testing. Modelling induced transient electrical fields in this way estimated a maximum voltage gradient of 74.35 nV cm/s^−1^ (Fig. 6) at the periphery of the tank, when a magnetic field change of 0.03 µT was presented. Sharks swimming through more than half of the radial area (from periphery to core) of the tank, at the time the charge was applied to the coil, would be exposed to transient electrical voltage gradients that previous experiments indicate were of a magnitude detectable via their electrosensory system^57^. Thus, we cannot definitively rule out the shark electrosense being involved in observed conditioned responses. Here we discuss the responses of conditioned animals tested under these circumstances. For reasons described below, we believe it to be unlikely that the transient voltage gradient acts as the stimulus or cue for the conditioned behavioural response.

In trials under control (unimpaired) conditions, the proportion of passes across the target, averaged per shark, was found to be significantly greater in the S+ minute (Fig. 1B) than any other one-minute time bin. This finding is unsurprising, as it replicates the findings reported by Meyer *et al*.^32^, although the applied magnetic field intensities in the Meyer *et al*. study were considerably higher than those applied in the present study. Nonetheless, response rates were comparable.

Perception thresholds of elasmobranchs to electrical stimuli have been well studied. Elasmobranch primary afferent (sensory neuron) response characteristics indicate adaptations to detection of weak phasic (sinusoidal) electrical fields near 1–2 hertz (Hz)^58^, such as those generated by ventilatory apparatus movement in prey. For an animal in the study to respond to any transient electrical artefact as a behavioural cue, it would need to distinguish the ‘signal’ of the induced electrical artefact from background electrical noise, as well as common-mode stimuli such as fields generated from its own ventilatory and osmoregulatory functions^41^. Habituation to electrical noise is possible, as long as it is a common-mode stimulus. Electrical noise in our experimental arena comes from multiple sources from within and outside of the building. Thus, it does not occur predictably, cyclically, or phasically. It is possible however, that once a source of electric field noise has become active, sharks in the experimental arena could habituate to some of this noise produced.

Key sources of random and unpredictable noise in this study come from electromagnetic interference (EMI), or radio-frequency interference (RFI), much of which is generated from the nearby airbase. These sources of noise are constant, but random and variable in both size and frequency. Thus, there is constant electrical noise in the tank from a variety of sources, with varying amplitude and frequency. It is very unlikely that the adaptive filter mechanism will suppress all this noise. Thus, entraining to the very small induced “spike” or electrical artefact seen when the coil is turned on is highly unlikely. The electrical artefact in the current experiments is small (maximum 74.35 nV/cm/s^−1^) not phasic, is very brief (2 milliseconds), and is presented against a complex electrical background comprised of e-fields several orders of magnitude greater (up to 1 mV/cm/s^−1^) than those generated by activation of the coil (Fig. 5). Again, entraining to the transient ‘signal’ generated by coil activation as a behavioural cue would be very challenging.

Given the constant noisy electrical background described, it could be expected that if the electrosensory system was solely responsible for detecting magnetic fields, a reduction in behavioural response would occur when presented with such weak magnetic stimuli and associated electrical transients. We did not observe any such diminished response with unimpaired animals. We cannot definitively conclude that the responses observed resulted solely from perception of the magnetic stimulus rather than perception of any transient electrical artefact. We can, however, still invoke the hypothesis that the ampullae of Lorenzini and the shark electrosensory system may not be the sole sensory receptor structures used to perceive magnetic field stimuli. The magnitude of magnetic stimuli presented in these experiments is within the range of values that Klimley^59^ hypothesised for navigation via magnetic fields in scalloped hammerhead sharks, *Sphyrna lewini* (Griffith and Smith, 1834), found in association with the Espirito Santo seamount complex in the Gulf of California. Klimley mapped the associated magnetic gradients in this region and found there to be less than a 50 nT range between magnetic maxima and minima. These data lend support to Klimley’s hypothesis that sharks can navigate via geomagnetic topotaxis. Further, our modifications to the vertical component of the local magnetic field affected the intensity or strength of the field within the tank, but did not affect magnetic field polarity (north-south directionality of the magnetic field). According to the principles of the active mode of induction proposed by Kalmijn^31, 33^, Paulin^28^, and Molteno & Kennedy^43^, it is the horizontal (polarity) component of the earth’s magnetic field that induce vertical electric fields that could convey information regarding magnetic field directionality. The induction based magnetoreceptor system is used to gain a compass heading regarding direction of travel, whereas direct magnetoreceptor mechanisms (e.g. a magnetite based system) are proposed to facilitate detection of anomalies or changes in magnetic field intensity, as occurred in this study. Our results therefore lend further support to hypotheses of non-electrosensory mediated magnetoreception.

An alternative hypothesised mechanism is magnetite-based magnetoreception, based on the presence of single-domain biogenic magnetite (Fe_3_O_4_) within specific tissues. These ferromagnetic crystals are proposed to form chains within cells, and changes in the ambient magnetic field directs the orientation of these chains. Clusters or chains of such crystals are necessary, as opposed to individual crystals, as this prevents oscillation of individual crystals in response to background thermal energy. Such structures have been described in some teleost and avian species^8, 23, 24, 60^. We hypothesised that elasmobranchs may be able to discriminate magnetic field intensities via a system homologous to that exhibited in some teleosts and birds. Thus, sensory impairment trials were incorporated into the study to further elucidate the existence of such structures.

Neodymium magnets embedded into gelatine filled, sealed containers were used to functionally block shark putative magnetoreceptor structures by creating a constant source of magnetic noise in the region of putative magnetoreceptor structures (in this case, putative structures housed within the olfactory organs). Encasing the magnets protects them from any galvanic action or electrochemical reactions arising from contact with the seawater, which may cause irritation to sharks in the form of overwhelming the electrosensory system, or a localized change in pH^47, 52^.

Sharks that had undergone impairment treatment were observed to swim normally, and feed readily with the magnets in place, without presentation of the magnetic stimulus. Normal behaviours of sharks in the tank were thus judged to not be affected by magnetic impairment treatment.

To ensure any change in response rates was due to the magnetic noise created by the impairment devices, rather than being a function of handling or application of the boxes containing the magnets, impaired sharks were presented with higher intensity magnetic stimulus (8–1.2 μT) but were not fed or given a food reward, *per* the protocol used in testing. We observed strong conditioned responses by impaired sharks when presented with stronger magnetic stimuli (8–5.3 μT) (Table 3), by comparison, weaker conditioned responses were observed when the magnetic stimulus was reduced (1.2–1.4 μT) (Table 3). Impairment treatment was judged to not effect response rates at higher applied magnetic intensities. This indicated magnetic flux and noise created by the neodymium magnets was not sufficient to mask stronger stimuli. Our observations during these initial trials indicated that application of the magnet containing boxes to the heads of sharks, and the physical weight of the magnets within boxes had no adverse effect on normal behaviours, or conditioned responses. However, we did not conduct comparative control trials with empty boxes attached. Thus, we cannot rule out the possibility that despite our observations and data pointing to the contrary, the presence of the boxes themselves (rather than their contents) may have acted as a chronic irritant that influenced conditioned responses over time.

A series of ten trials was carried out to compare conditioned responses of impaired sharks with the results of trials using unimpaired sharks (magnetic stimulus = (0.03 μT). Median response (median number of passes over the target by all sharks combined) during stimulus presentation in sensory impaired trials was 2 (Fig. 3A), compared with 13.5 in unimpaired trials (Fig. 1A). Again, to ensure validity of our findings, the proportion of passes across the target, averaged per shark, per one minute time bin, for each of the ten trials was calculated. Sharks with magnetic impairment showed no significant statistical difference the proportion of passes across the target, averaged per shark, across all 21 of the one-minute time bins over the ten-trial series (Fig. 3B).

Studies into the repellent properties of permanent magnets have shown a density effect, where a positive relationship was found between the number of sharks and the depredation of baits^47–49^. These studies have also demonstrated that sharks quickly habituate to the repellent effect of permanent, that may be caused by irritation^47, 52^. A minimum of three sharks was required to elicit this effect^48^. In our impairment trials, the minimum number of sharks used was three (in 3 of 10 trials), four sharks were used in the remaining 7 trials. Reduced responses seen (averaged per shark) are therefore not considered to be related to the presence of fewer sharks compared with the control trials. In-fact, in our unimpaired trials, the maximum responses seen were not in the two trials with 7 sharks. Galvanic action that may cause irritation was prevented in our experiments through encasing the magnets.

The results of the trials with impaired sharks (those with magnets attached) may lend further evidence that the observed conditioned responses were not mediated by the electrosensory system. There is no *a priori* reason to believe that the head-mounted magnets interfered with any voltage transients induced by coil activation, thus we consider the cue for the conditioned behaviours seen in the impairment experiments were magnetic field changes *per se* rather than to electrical artefacts. We observed impaired animals produce a strong conditioned response under a stronger (8µT) magnetic stimulus, and a reduced conditioned response under a reduced (0.03 µT) magnetic stimulus. If irritation were the cause for a reduced response, one might expect it would be seen under the stronger stimulus presentation too, which it was not.

Johnsen and Lohmann^44^ commented that the results of magnetic impairment studies by Walker^46^ did not account for the possibility that the magnets used induced an electrical signal through lag in the movement of the magnets relative to the head. The magnetic noise created by our impairment methods serves to mask the magnetic signal presented during the S+ condition in testing, decreasing the chance of the conditioned response being produced. We postulate that contrary to the concerns raised by Johnsen and Lohmann^44^, it is unlikely that magnetic noise created by the impairment methods interferes with the ability of the shark electrosensory system to determine changes in the background electrical field. The electrosensory adaptive filter mechanism is central to the sensitivity of the shark electrosense to very small voltage gradients.

It is not known if such an adaptive filter exists for magnetic field stimuli perceived via any non-electrosensory means. Magnetic noise created by the magnets placed on the head of a shark induces an electrical field in much the same way as the body of the shark does as it swims through the ambient magnetic field. The placement of the magnets in gelatine can be likened to the function of the shark otoconia within the gelatinous cupula of the vestibular sensory maculae. The magnets are heavier than their surrounding medium, and their movement will lag slightly behind the movement of the head. The induced electric field generated by the attached magnets as the shark swims is characteristically phasic due to the sinusoidal movement of the shark’s head, and thus becomes a common mode stimulus that would be effectively negated by the adaptive filter mechanism. When a change to that phasic pattern occurs (i.e. when a transient voltage gradient is generated through presentation of the magnetic field in the S+ minute), the induced electrical signal that change creates is more prominent (signal to noise ratio is higher). Such a signal should be no less discernable than if the magnets were not attached. Thus, when the magnetic stimulus is presented, perception of the resulting induced electrical transient would not be impaired by the magnets placed on the sharks’ head.

These data, in combination with the arguments we have set out, lend support to the existence of a non-electrosensory/induction-based magnetoreceptor structure capable of perceiving changes in magnetic field intensity. The placement of the magnets was designed to test the hypothetical existence of an olfactory based magnetoreceptor in elasmobranch fishes that functions in a homologous manner to that described in some teleost and avian species, as has been proposed previously^25, 61, 62^. Our results cannot definitively support the existence and use of such a magnetoreceptor. They do, however, provide support to hypotheses that the electrosensory system of sharks may not be the sole means by which they are able to detect magnetic stimuli.

We conclude that it is likely the diminished responses seen under magnetic impaired conditions were as a result of impairment to a non-electrosensory magnetoreceptor structure. This conclusion could, however, be further supported through incorporation of further experimental replicates with sham magnets (inert objects of the same approximate size and weight). Sharks used in the impairment study produced the CR when presented with stronger magnetic stimuli than used in unimpaired testing, and showed no observable change in ‘normal’ behaviours. We therefore consider it very unlikely that the diminished response seen in testing under weak magnetic stimuli using sensory-impaired sharks was an artefact of the placement of the boxes containing the magnets, or were due to the weight of the magnets, rather than being due to the magnetic fields that stem from the magnets themselves. Thus, there is no *a priori* reason for the attachment of the boxes, or the weight of the contents of the boxes to affect the sharks’ reactions to the magnetic stimuli used in testing. However, as we did not incorporate this control group into our study, we must interpret our results with a degree of caution.

While not definitive, these data present a platform for future study to further elucidate which sensory structures are involved, and which are the neural pathways relaying information to the brain. It is unlikely that sharks possess only one modality or mechanism for detection of magnetic fields, as has been suggested^63^. Indeed, the majority of vertebrates and invertebrates either hypothesised or demonstrated to respond to magnetic field stimuli do not have an electrosense, and other theories have been proposed as to the means for detection of magnetic stimuli. Whether such mechanisms have arisen through convergent or divergent evolutionary processes also remains the subject of debate. These mechanisms include a light governed chemical reaction based either on the radical pair hypothesis^64–66^, or a pineal window/light based magnetoreception hypothesis^15, 67–69^.

The magnetite based system has been argued to be the ancestral means by which magnetoreception has arisen across taxa^61, 63^, including the elasmobranch fishes^61^, thus other proposed or demonstrated systems are argued to be more derived. Migratory birds are postulated to make use of the both a magnetite based receptor system, as well as a light based/radical pairs mechanism in photoreceptors^70^. It is equally possible that sharks possess the capability to perceive the different parameters of the geomagnetic field via different physical mechanisms.

Light-dependent models of magnetoreception are proposed to involve an interaction between the magnetic field and either magnetite particles located within a photoreceptor or excited states of photopigment molecules^14^. The models require polarized light, making this system less likely a source of constant magnetic field information in elasmobranchs. In percomorph teleosts the threshold saturation for detection of polarized light is 60%. At 2 metres below the surface in pelagic waters, polarized light saturation is ~40%^71^. Thus, unless an animal is swimming within the first two metres of the water column, it would not receive sufficient irradiation required by light based mechanisms. It should be noted however that crepuscular periods offer optimal polarized light saturation (63%)^71^, thus could aid in explaining crepuscular “spike dive” behaviour seen in many pelagic fish species, including sharks.

Further studies into magnetic sensory capabilities in sharks are needed to further elucidate these mechanisms. Of course, the ability to perceive a sensory stimulus does not confer the use of that sensory capability in a functional role. Thus, further studies should seek to qualify and quantify roles and functions of different putative sensory systems/structures, and should test the ecological validity of such hypotheses.

## Materials and Methods

We adapted the protocol of Meyer *et al*.^32^ to condition sharks to respond to a modification of the local magnetic field. Captive Sandbar sharks were housed in a 7 m diameter circular tank, surrounded by 100 turns of 18 AWG copper wire, spaced over a vertical distance of 120 cm. In training and conditioning, a charge ranging from 12 V DC to 1.5 V DC with a 5.6k Ω resistor was applied to the coil, producing a localised magnetic field that varied in total intensity from 2.8 μT to 0.029 μT respectively (Fig. 1). In testing trials, a 1.5 V DC charge with a 5.6k Ω resistor was used, to modify the local magnetic field within the experimental arena by 0.029 μT.

### Animal Training and Testing

Training and test trials were carried out over a six-month period. A maximum of two weeks was allowed for training of new sharks. Up to seven animals were held and trained together during training and testing phases, as social learning in sharks has been demonstrated to be faster and more effective in behavioural studies than training novice animals alone^72, 73^. To produce the conditioned response, captive sharks were presented with a food reward over an 80 × 80 cm target every time the magnetic field was modified, field modification in training varied pseudo-randomly, ranging between 2.8 μT to 0.029 μT. Animals were fed a ration of food this way every day over the course of the study period except during testing phases. To maximise feeding motivation, sharks were not fed for 24 hours prior to testing. To ensure that observed responses (convergence upon the target) were due to the conditioning protocol used, sharks in the study were not given a food reward during test trials.

Ten test trials were carried out under both unimpaired (normal) and magnetosensory impaired conditions (see *Sensory Impairment* subsection for description of impairment methods). Behavioural trials were carried out pseudo-randomly over the last four months of the six-month experimental period, with continued reinforcement training between trials. Following the methodology of Meyer *et al*.^32^, each trial duration lasted 21 minutes, during which time shark behaviours were observed over a time-series comprised of 10 minutes observation under normal local/background magnetic field (S−), followed by a one minute modification of the local magnetic field (S+), followed by a further 10 minutes observation (S−). *Per* Meyer *et al*.^32^, counts of the total number of passes by all sharks over the target in each trial were pooled into one-minute time bins, across all trials.

Animals that became too aggressive, or were judged to disrupt normal conditioned behaviours by dominating or outcompeting other sharks were removed from the study once replacement animals had been trained. Under unimpaired conditions, five sharks were used for the first eight trials. Two sharks were subsequently added to the group for training, prior to the removal of two sharks from the existing pool that were judged to outcompete other sharks and disrupt conditioned behaviours. Two trials were completed with seven sharks (Table 2). The increased number of sharks did not appear to affect the overall number of passes over the target during stimulus presentation (see results and Table 2), thus no further trials were run under control conditions.

### Sensory Impairment

To induce magnetoreceptor impairment in test trials, 1.2 × 0.3 × 0.3 cm neodymium magnets (Apex Magnets, Petersburg, WV, USA) were placed in gelatine filled 2.5 × 2.5 × 1 cm plastic boxes which were temporarily (and reversibly) attached to the heads of the test animals following behavioural conditioning. The magnets were embedded horizontally within the gelatine and the boxes sealed. The gelatine serves as a semi-liquid matrix, allowing small movement of the magnets in conjunction with movement of the animal, thus maintaining a small but constant magnetic flux. Sealing the containers prevented any contact between the magnet and sea water, eliminating the possibility of electrical currents forming as a result of galvanic action. Maximal magnetic flux over putative magnetoreceptor structures was achieved through alignment of the longitudinal axes of the magnets and the olfactory organs (Fig. 2). Thus, the magnetic flux created generated a constant source of magnetic ‘noise’. These boxes were glued to the skin of the shark over the dorsal surface of olfactory capsules. Sharks continued behavioural reinforcement/training under variable strength magnetic field modification, as per the methods used in the control series.

To ensure that sharks that had undergone impairment treatment were capable of producing the conditioned response (CR), five trials were conducted at higher presented magnetic field strengths (8–1.2μT) (Table 3). These initial tests followed the same protocol as that used in control/unimpaired testing (trials carried out pseudo-randomly, animals not fed during testing, constant training/reinforcement with food reward between trials).

Five sharks were used in initial training and testing in the magnetic impaired series (Table 3). However, two sharks were removed from the study prior to commencement of trials at 0.03µT, both of which had grown considerably larger than, and outcompeted the remaining three sharks. Two trials at 0.03µT were initially run with three sharks, before a fourth shark was added to the test group. When this shark had been sufficiently conditioned, a further seven trials were run using four sharks. One further shark was removed (due to the same reasons) for the remaining trial.

Sharks in both the unimpaired and impaired groups were trained and tested together, with the exception of the final shark added in the impaired trial series, which was trained but not tested under control conditions.

All experiments were approved by the University of Hawaii Institutional Animal Care Advisory Committee (IACUC), protocol # 13–1749. All methods used were in accordance with the approved protocol, and the IACUC guidelines set out.

### Data Collection and Analysis

A total of 20 trials were carried out under control/normal (unimpaired, n = 10) and experimental (sensory impaired, n = 10) conditions. Each trial was recorded remotely from an aerial perspective for subsequent analysis using a high-resolution video camera. Sharks in the study were tested as a group, as count information was not available on an individual level, due to the difficulty in reliably identifying individual sharks in each trial across the series. Thus, counts of total passes over the target by all sharks, in each minute of each trial were pooled into one-minute time bins. The median number of passes and the standard error of the mean for each one-minute time bin across all ten trials under both experimental conditions (unimpaired/impaired) was subsequently calculated.

The number of sharks used in testing was not constant across either series of trials (unimpaired/impaired), thus, to standardize our data we calculated the proportion of passes across the target, averaged per shark, per minute, for every trial, in each series. Our data did not follow a normal distribution, thus, Friedman Rank Sum tests and Wilcoxon Signed Rank post-hoc analysis were used to discriminate any differences in mean proportion of passes, per shark, between time bins and to identify where differences arose for each trial series (unimpaired/impaired).

Wilcoxon Rank Sum tests were used to compare mean proportion of passes across the target, per shark, in the 11^th^ minute (S+) time-bin under normal and impaired conditions.

### Characterising the Magnetic and Electric Fields

Modifications to the local magnetic field were measured using a MR3 Milligauss Meter (AlphaLab Inc., Salt Lake City, UT, USA). Measurement of any induced transient electrical artefact in the uniform electrical field associated with supplying power to the coil was measured using a Trifield^®^ Natural EM Meter (AlphaLab Inc., Salt Lake City, UT, USA). Equipment normally used in electrophysiological experiments was also modified to measure transient voltage gradients. Nonpolarizable Ag-AgCl half-cell electrodes (World Precision Instruments Inc, Sarasota, Fl) were fitted to agar-filled capillary tubes that were immersed in the water in different locations throughout the test arena. The output from the two electrodes was differentially amplified (DP-304; Warner Instruments) at 1000x–10,000x, filtered at 60 Hz (Hum Bug, Quest Scientific, Vancouver, British Columbia), digitized on a recording oscilloscope (Tektronix Inc, Beaverton, Or).

### Electric Field Calculation

The induced electrical field (Volts m) at any point in time, at any point in the tank can be calculated from Faraday’s Law of Induction:

1 $${\varepsilon }_{ind}=\,{\oint }^{}{\overrightarrow{E}}_{ind}\cdot d\overrightarrow{s}=-\frac{d}{dt}({{\rm{\Phi }}}_{{\rm{B}}})$$

whereby the induced electrical field (ε_ind_) within the coil is equal to the negative time rate of change of the rate of magnetic flux ($-\frac{d}{{dt}}({{\rm{\Phi }}}_{{\rm{B}}})$).

2 $${\oint }^{}\overrightarrow{E}\cdot d\overrightarrow{s}=2\pi r|{\rm{{\rm E}}}|$$

Thus, we can calculate ε_ind_ around the whole circuit, using a line integral, summing the EMF produced at each and every point over the length of the wire (coil).While

3 $$\frac{d}{dt}({{\rm{\Phi }}}_{{\rm{B}}})=\frac{d}{dt}\,(B\,A)$$

ε_ind_ is equal to the time rate of change magnetic flux, which is equal to the time rate of change of the product of the perpendicular component of the magnetic field (*B*) and the area inside the coil (*A*).

4 $$=\frac{d}{dt}(B\,\pi \,{r}^{2})$$

5 $$=\pi {r}^{2}{\mu }_{^\circ }\frac{d}{dt}(I(t))$$

Alternatively, ε_ind_ can expressed as formula 5; the area inside the coil multiplied by the time rate of change of the current in the circuit ($\frac{d}{{dt}}(I(t))$), where ($\mu ^\circ$) is the magnetic permeability constant according to Biot-Savart law.This can be simplified to formula 6.

6 $$=\pi {r}^{2}\cdot {\mu }_{^\circ }b$$

where (*b*) is a constant equal to $\frac{d}{{dt}}(I(t))$.

Therefore, using Faraday’s Law in general form:

7 $$2\pi r{\rm{{\rm E}}}=\pi {r}^{2}\cdot {\mu }_{^\circ }b$$

thus

8 $${\rm{E}}=\frac{(-\pi {r}^{2}{\mu }_{^\circ }b)}{2\pi r}$$

where r is the distance (metres) from the axis of the coil (the centre of the tank).

Change or flux in the magnetic field within the tank occurs during the time taken for the current moving through the circuit (coil) to reach its’ maximum value. To determine *d*t, it is first necessary to calculate inductance within the coil. As the length (height) of the coil is not greater than its diameter, it is not appropriate to use standard equations for coil inductance. Instead, it is appropriate to model inductance using Wheelers’ formula (11)^74^, which improves upon the equation put forward by Nagaoka^75^.

9 $${L}_{s}=0.002\pi d{N}^{2}[\mathrm{ln}(1+\frac{\pi d}{2\ell })+\,\frac{1}{2.3004+\frac{3.2\ell }{d}+1.7636{(\frac{\ell }{d})}^{2}}]$$

where L_S_ is in µH; d= coil diameter (cm); ℓ = coil height (cm); N = number of turns.

Having calculated inductance, we then modelled current flow in the circuit using the LTspice IV (Linear Technology, CA, USA) modelling program, to determine *d*t (time taken for current in the circuit to reach its steady-state value). However, we must express ε_ind_ as a function of time (as ε_ind_ is dependent upon magnetic flux, and magnetic flux is dependent upon the time taken to reach steady state value, i.e. the time taken for current in the circuit to reach a steady state) (formula 9).

10 $${\varepsilon }_{ind}({\rm{t}})=\frac{r}{2}\,.{\mu }_{^\circ }b$$

r = distance (metres) from the axis of the coil (the centre of the tank).

Finally, we can account for the specific physical characteristics of the coil used in our experiments; namely determining the number of turns of the coil per unit height.

11 $${\varepsilon }_{ind}({\rm{t}})=\,\frac{{\mu }_{^\circ }{\rm{rNb}}}{2h}$$

where ${{\rm{\mu }}}_{^\circ }$ is the magnetic permeability of the medium, r is the distance (metres) from the axis of the coil (the centre of the tank), N is the number of turns of the coil, b is time rate of change of the current in the circuit to reach a steady state [$\frac{d}{{dt}}(I({\rm{t}}))]$, *h* is the height of the coil.

### Data availability

The datasets generated during and/or analysed during the current study are available from the corresponding author on reasonable request.

## References

[1] Wiltschko R, Wiltschko W (2006). Magnetoreception. Bioessays.

[2] Mann S, Sparks N, Board R (1990). Magnetotactic bacteria: microbiology, biomineralization, palaeomagnetism and biotechnology. Adv. Microb. Physiol..

[3] Blakemore R (1975). Magnetotactic bacteria. Science.

[4] Torres FF, Botanica D, Paulo S (1986). Magnetite magnetotaxis algae. Biophys. J..

[5] Brown F, Webb H, Barnwell F (1964). A compass directional phenomenon in mud-snails and its relation to magnetism. Biol. Bull..

[6] Lohmann K (1995). Magnetic orientation of spiny lobsters in the ocean: experiments with undersea coil systems. J. Exp. Biol..

[7] Hsu C-Y, Ko F-Y, Li C-W, Fann K, Lue J-T (2007). Magnetoreception system in honeybees (Apis mellifera). PLoS One.

[8] Beason R, Nichols J (1984). Magnetic orientation and magnetically sensitive material in a transequatorial migratory bird. Nature.

[9] Wiltschko W, Wiltschko R (1972). Magnetic compass of European robins. Science.

[10] Thalau P, Ritz T, Burda H, Wegner RE, Wiltschko R (2006). The magnetic compass mechanisms of birds and rodents are based on different physical principles. J. R. Soc. Interface.

[11] Wiltschko W, Dehe L, Stapput K, Thalau P, Wiltschko R (2010). Magnetoreception in birds: no intensity window in ‘fixed direction’ responses. Naturwissenschaften.

[12] Marhold S, Wiltschko W, Burda H (1997). A magnetic polarity compass for direction finding in a subterranean mammal. Naturwissenschaften.

[13] Deutschlander M, Borland S, Phillips J (1999). Extraocular magnetic compass in newts. Nature.

[14] Deutschlander ME, Phillips JB, Borland SC (2000). Magnetic Compass Orientation in the Eastern Red-Spotted Newt, Notophthalamus viridescens: Rapid Acquisition of the Shoreward Axis. Copeia.

[15] Freake M, Phillips J (2005). Light Dependent Shift in Bullfrog Tadpole Magnetic Compass Orientation: Evidence for a Common Magnetoreception Mechanism in Anuran and Urodele Amphibians. Ethology.

[16] Lohmann K, Lohmann C (1994). Detection of Magnetic Inclination Angle By Sea Turtles: a Possible Mechanism for Determining Latitude. J. Exp. Biol..

[17] Light P, Salmon M, Lohmann K (1993). Geomagnetic orientation of loggerhead sea turtles: evidence for an inclination compass. J. Exp. Biol..

[18] Kirschvink JL, Dizon AE, Westphal JA (1986). Evidence from Strandings for Geomagnetic Sensitivity in Cetaceans. J. Exp. Biol..

[19] Durif CMF (2013). Magnetic Compass Orientation in the European Eel. PLoS One.

[20] Mora CV (2014). Behavioral modification of visually deprived lemon sharks (Negaprion brevirostris) towards magnetic fields. J. Navig..

[21] Mora CV, Davison M, Walker MM (2009). Conditioning as a Technique for Studying the Sensory Systems Involved in Animal Orientation, Homing and Navigation – a Review. J. Navig..

[22] Walker M (1984). Learned magnetic field discrimination in yellowfin tuna,Thunnus albacares. J. Comp. Physiol. A.

[23] Diebel CE, Proksch R, Green CR, Neilson P, Walker MM (2000). Magnetite defines a vertebrate magnetoreceptor. Nature.

[24] Walker MM (1997). Structure and function of the vertebrate magnetic sense. Nature.

[25] Walker, M. M., Diebel, C. E. & Kirschvink, J. L. In *Sensory Processing in Aquatic Environments* 53–74 (Springer-Verlag, 2004).

[26] Quinn TP (1980). Evidence for celestial and magnetic compass orientation in lake migrating sockeye salmon fry. J. Comp. Physiol. A.

[27] Quinn TP, Brannon EL (1982). The use of celestial and magnetic cues by orienting sockeye salmon smolts. J. Comp. Physiol. A.

[28] Paulin M (1995). Electroreception and the compass sense of sharks. J. Theor. Biol..

[29] Akoev G, Ilyinsky O, Zadan P (1976). Responses of electroreceptors (ampullae of Lorenzini) of skates to electric and magnetic fields. J. Comp. Physiol. A.

[30] Brown H, Ilyinsky O (1978). The ampullae of Lorenzini in the magnetic field. J. Comp. Physiol..

[31] Kalmijn A (1981). Biophysics of geomagnetic field detection. IEEE Trans. Magn..

[32] Meyer CG, Holland KN, Papastamatiou YP (2005). Sharks can detect changes in the geomagnetic field. J. R. Soc. Interface.

[33] Kalmijn A (1982). Electric and magnetic field detection in elasmobranch fishes. Science.

[34] Andrianov GN, Brown HR, Ilyinsky OB (1974). Responses of central neurons to electrical and magnetic stimuli of the ampullae of lorenzini in the Black Sea skate. J. Comp. Physiol. A.

[35] Bonfil R (2005). Transoceanic migration, spatial dynamics, and population linkages of white sharks. Science.

[36] Weng KC (2007). Migration and habitat of white sharks (Carcharodon carcharias) in the eastern Pacific Ocean. Mar. Biol..

[37] Papastamatiou YP (2011). Scales of orientation, directed walks and movement path structure in sharks. J. Anim. Ecol..

[38] Papastamatiou YP (2013). Telemetry and random-walk models reveal complex patterns of partial migration in a large marine predator. Ecology.

[39] Kalmijn A (2000). Detection and processing of electromagnetic and near-field acoustic signals in elasmobranch fishes. Philos. Trans. R. Soc. Lond. B. Biol. Sci..

[40] Montgomery JC (1980). Dogfish horizontal canal system: responses of primary afferent, vestibular and cerebellar neurons to rotational stimulation. Neuroscience.

[41] Montgomery J, Bodznick D (1993). Hindbrain circuitry mediating common mode suppression of ventilatory reafference in the electrosensory system of the little skate Raja erinacea. J. Exp. Biol..

[42] Montgomery J, Bodznick D (1999). Signals and noise in the elasmobranch electrosensory system. J. Exp. Biol..

[43] Molteno TCA, Kennedy WL (2009). Navigation by induction-based magnetoreception in elasmobranch fishes. J. Biophys..

[44] Johnsen S, Lohmann KJ (2005). The physics and neurobiology of magnetoreception. Nat. Rev. Neurosci..

[45] Johnsen, S. & Lohmann, K. J. Magnetoreception in animals. (2008).

[46] Walker, M., Diebel, C. E. & Kirschvink, J. L. In *Fi*sh Physiol*og*y: Se*nsory Systems Neuroscience* (eds Hara, T. J. & Zielinski, B.) **25**, 335–374 (Academic Press, 2006).

[47] Brill R (2009). The repulsive and feeding-deterrent effects of electropositive metals on juvenile sandbar sharks (Carcharhinus plumbeus). Fish. Bull.

[48] Robbins WD, Peddemors VM, Kennelly SJ (2011). Assessment of permanent magnets and electropositive metals to reduce the line-based capture of Galapagos sharks, Carcharhinus galapagensis. Fish. Res..

[49] O’Connell CP, Abel DC, Gruber SH, Stroud EM, Rice PH (2011). Response of juvenile lemon sharks, Negaprion brevirostris, to a magnetic barrier simulating a beach net. Ocean Coast. Manag..

[50] O’Connell, C., Abel, D., Stroud, E. & Rice, P. Analysis of permanent magnets as elasmobranch bycatch reduction devices in hook-and-line and longline trials. *Fish. Bull*. 394–402 (2011).

[51] Hutchinson M (2012). The effects of a lanthanide metal alloy on shark catch rates. Fish. Res..

[52] McCutcheon SM, Kajiura SM (2013). Electrochemical properties of lanthanide metals in relation to their application as shark repellents. Fish. Res..

[53] Newton, K. C. & Kajiura, S. M. Magnetic field discrimination, learning, and memory in the yellow stingray (Urobatis jamaicensis). *Anim. Cogn*. doi:10.1007/s10071-017-1084-8 1–12 (2017). 10.1007/s10071-017-1084-8 28343270

[54] Siegenthaler A, Niemantsverdriet PRW, Laterveer M, Heitkönig IMA (2016). Aversive responses of captive sandbar sharks Carcharhinus plumbeus to strong magnetic fields. J. Fish Biol..

[55] Mora C, Davison M, Wild J, Walker M (2004). Magnetoreception and its trigeminal mediation in the homing pigeon. Nature.

[56] Kajiura SM, Holland KN (2002). Electroreception in juvenile scalloped hammerhead and sandbar sharks. J. Exp. Biol..

[57] Bedore CN, Kajiura SM (2013). Bioelectric fields of marine organisms: voltage and frequency contributions to detectability by electroreceptive predators. Physiol. Biochem. Zool..

[58] Tricas TC, New JG (1998). Sensitivity and response dynamics of elasmobranch electrosensory primary afferent neurons to near threshold fields. J. Comp. Physiol. A..

[59] Klimley AP (1993). Highly directional swimming by scalloped hammerhead sharks, Sphryna lewini, and subsurface irradiance, temperature, bathymetry and geomagnetic field. Mar. Biol..

[60] Beason R, Semm P (1996). Does the avian ophthalmic nerve carry magnetic navigational information?. J. Exp. Biol..

[61] Kirschvink JL, Walker MM, Diebel CE (2001). Magnetite-based magnetoreception. Curr. Opin. Neurobiol..

[62] Mora, C. V., Davison, M., Wild, J. M. & Walker, M. M. Magnetoreception and its trigeminal mediation in the homing pigeon. *Nature* **432**, (2004). 10.1038/nature03077 15565156

[63] Gould JL (2008). Magnetoreception. Curr. Biol..

[64] Schulten K, Swenberg CE (1978). A biomagnetic sensory mechanism based on magnetic field modulated coherent electron spin motion. *für Phys*. Chemie.

[65] Ritz T, Adem S, Schulten K (2000). A model for photoreceptor-based magnetoreception in birds. Biophys. J..

[66] Mouritsen H, Ritz T (2005). Magnetoreception and its use in bird navigation. Curr. Opin. Neurobiol..

[67] Gruber S, Hamasaki D, Davis B (1975). Window to the epiphysis in sharks. Copeia.

[68] Semm P, Demaine C (1986). Neurophysiological properties of magnetic cells in the pigeons visual-system. J. Comp. Physiol. A.

[69] Phillips JB, Deutschlander ME, Freake MJ, Borland SC (2001). The role of extraocular photoreceptors in newt magnetic compass orientation: parallels between light-dependent magnetoreception and polarized light detection in vertebrates. J. Exp. Biol..

[70] Wiltschko W, Wiltschko R (2005). Magnetic orientation and magnetoreception in birds and other animals. J. Comp. Physiol. A..

[71] Novales Flamarique I, Hawryshyn CW (1997). Is the use of underwater polarized light by fish restricted to crepuscular time periods?. Vision Res..

[72] Guttridge TL (2013). Social learning in juvenile lemon sharks, Negaprion brevirostris. Anim. Cogn..

[73] Guttridge TL, Brown C (2014). Learning and memory in the Port Jackson shark, Heterodontus portusjacksoni. Anim. Cogn..

[74] Wheeler H (1982). Inductance Formulas for Circular and Square Coils. Proc. IEEE.

[75] Nagaoka H (1909). The Inductance Coefficients of Solenoids. Journal of the College of Science, Imperial University.

[76] Baumgartner J (2013). Magnetotactic bacteria form magnetite from a phosphate-rich ferric hydroxide via nanometric ferric (oxyhydr)oxide intermediates. Proc. Natl. Acad. Sci. USA.

[77] Prato FS, Kavaliers M (1996). Behavioural responses to magnetic fields by land snails are dependent on both magnetic field direction and light. Proc. Biol. Sci..

[78] Phillips J (1986). Two magnetoreception pathways in a migratory salamander. Science.

[79] Phillips J, Jorge P, Muheim R (2010). Light-dependent magnetic compass orientation in amphibians and insects: candidate receptors and candidate molecular mechanisms. J. R. Soc. Interface.

